# Patterns of Opioid and Non-Opioid Analgesic Consumption in Patients with Post-COVID-19 Conditions

**DOI:** 10.3390/jcm12206586

**Published:** 2023-10-18

**Authors:** Pilar Carrasco-Garrido, Domingo Palacios-Ceña, Valentín Hernández-Barrera, Isabel Jiménez-Trujillo, Carmen Gallardo-Pino, Cesar Fernández-de-las-Peñas

**Affiliations:** 1 Department of Medical Specialties and Public Health, Health Sciences Faculty, Universidad Rey Juan Carlos, Avenida Atenas s/n, Alcorcon, 28922 Madrid, Spain; valentin.hernandez@urjc.es (V.H.-B.); isabel.jimenez@urjc.es (I.J.-T.); carmen.gallardo@urjc.es (C.G.-P.); 2 Preventive Medicine and Public Health Teaching and Research Unit, Health Sciences Faculty, Rey Juan Carlos University, Avda. Atenas s/n. Alcorcón, 28922 Madrid, Spain; 3 Department of Physical Therapy, Occupational Therapy, Rehabilitation and Physical Medicine, Health Sciences Faculty, Universidad Rey Juan Carlos, Avenida Atenas s/n, Alcorcon, 28922 Madrid, Spain; domingo.palacios@urjc.es (D.P.-C.); cesar.fernandez@urjc.es (C.F.-d.-l.-P.)

**Keywords:** long-COVID, opioids, non-opioids, predictors

## Abstract

Pain is a major health issue for healthcare systems, and access to pain treatment is a fundamental human right. Pain is a common symptom experienced in the post-COVID phase by a significant percentage of patients. This study describes the prevalence and associated factors associated with the use of opioid and non-opioid analgesics in subjects with post-COVID-19 condition. Sociodemographic data, post-COVID symptoms, health profile, and opioid and non-opioid analgesic consumption were collected in 390 subjects with post-COVID-19 condition. We analyzed the independent effect of all variables on opioid/non-opioid analgesic consumption by using logistic multivariate regressions. The prevalence of opioid and non-opioid analgesic consumption was 24.1% and 82.3%, respectively. Tramadol (17.18%) and codeine (7.95%) were the most commonly used opioid analgesics, and Paracetamol (70%) and ibuprofen (45.4%) were the most commonly used non-opioid analgesics. Females were more likely to consume non-opioid analgesics (aOR2.20, 95%CI 1.15, 4.22) than males. Marital status of married/partner vs. single (aOR2.96; 95% CI 1.43, 6.12), monthly income < EUR 1000 VS. > EUR 2000 (aOR3.81; 95% CI 1.37, 10.61), number of post-COVID symptoms < 5 (aOR2.64, 95%CI 1.18, 5.87), and anxiolytics consumption (aOR 1.85, 95%CI 1.05, 3.25) were associated with a greater likelihood of opioid analgesic consumption. Age > 55 years (aOR3.30, 95%CI 1.34, 8.09) and anxiolytics consumption (aOR2.61, 95%CI 1.36, 4.98) were associated with a greater likelihood of non-opioid analgesic consumption. Opioid analgesic consumption was highly associated (aOR 3.41, 95%CI 1.27, 6.11) with non-opioid analgesic consumption. The prevalence of opioid analgesic and non-opioid analgesic consumption in individuals with post-COVID-19 condition was 24.1% and 82.3%. Females with post-COVID-19 condition showed higher non-opioid analgesic consumption than men. Predictors of opioid consumption were marital status, lower monthly income, number of post-COVID symptoms, and anxiolytic consumption. Older age and anxiolytic consumption were predictors of non-opioid consumption.

## 1. Introduction

Patients with a Severe Acute Respiratory Syndrome Coronavirus-2 (SARS-CoV-2) infection develop coronavirus disease 2019 (COVID-19) and exhibit a plethora of symptoms affecting the respiratory, cardiovascular, gastrointestinal, neurological, and musculoskeletal systems [1]. Musculoskeletal pain (myalgia) is a symptom at the acute phase of SARS-CoV-2 infection experienced by almost 25% of the patients [2,3]. Similarly, headache is also a COVID-19-associated onset symptom experienced by up to 30% of the patients [4,5].

Several patients who surpassed the acute phase of COVID-19 disease exhibit long-lasting symptoms (e.g., fatigue, dyspnea, pain, muscle weakness, depression, and persistent headache), which impair their quality of life. In fact, evidence suggests that between 20% and 40% of subjects who have survived COVID-19 develop a plethora of post-COVID symptoms during the first year after the infection [6,7]. The presence of long-lasting symptoms is called long-COVID [8] or post-COVID-19 condition [9]. Among long-lasting symptomatology, pain is a common symptom experienced in the post-COVID phase by almost 20% of the patients [10]. Studies focusing specifically on this symptom have revealed prevalence rates of post-COVID pain from 45% to 60% [11,12]. Similarly, post-COVID headache can be present alone or in combination with other long-lasting symptomatologies. Overall, post-COVID headache can affect 8% to 15% of COVID-19 survivors in the first 6 months after infection [13,14].

Pain is a major health issue for healthcare systems, and access to pain treatment is a fundamental human right. Due to the complexity of post-COVID-19 condition, individuals exhibiting post-COVID pain will require a multidisciplinary treatment approach, including pharmacological and non-pharmacological strategies, as well as physical rehabilitation and mental health and social services support [15].

One of the most important interventional issues for chronic pain management is the use of medication, particularly opioids and non-opioid analgesics. During the COVID-19 pandemic, there was an increase in internet searches searching for information on two common over-the-counter analgesics, ibuprofen and acetaminophen [16,17]. A study conducted by the Spine Intervention Society (SIS) revealed an increase in opioid prescriptions by up to 28.8% and a 26.2% increase in acetaminophen prescriptions by medical doctors during the first month of the COVID-19 outbreak [18].

A study using a nationwide commercial insurance database in the United States of America reported that opioid prescriptions likely increased during the early pandemic in 2020, whereas the prescription of non-pharmacological therapies decreased for individuals with chronic pain [19]. Similarly, opioid consumption increased from 19.79 DHD (defined daily dose/1000 inhabitants per day) in 2019 to 20.88 DHD in 2021 [20], whereas non-opioid analgesic consumption has risen from 32.90 DHD in 2019 to 40.05 DHD in 2021 [21] according to data reported by the Spanish Agency of Medicines and Health Products. An uncontrolled increase in opioid consumption could lead to opium abuse, which has been associated with higher mortality in hospitalized COVID-19 patients [22].

Despite this consumption increase in opioid and non-opioid analgesics, evidence about consumption patterns of opioid and non-opioid analgesics among patients with post-COVID-19 condition is yet lacking. The aim of this study was to investigate the prevalence and factors associated with opioid and non-opioid analgesic consumption in a Spanish population suffering from post-COVID-19 condition.

## 2. Methods

### 2.1. Study Design

A web-based cross-sectional survey design was used in the current pharmaco-epidemiological study. An online survey, including a total of 39 questions about post-COVID symptoms and the use of medication, was developed by the research team. The survey was uploaded to the Google Forms platform (Google LLC, Mountain View, CA, USA) and was open to be completed from 15 December 2021 to 15 March 2022.

### 2.2. Participants

Participants were recruited by social networks and internet platforms for COVID-19 patient support using the databases of two Spanish long-COVID organizations (*COVID persistente España* and *COVID persistente Madrid)*. Inclusion criteria for fulfilling the survey consisted of the following: (1) individuals who had survived SARS-CoV-2 acute infection; (2) were aged over 18 years old; and (3) were reporting long-lasting symptoms for at least three months after SARS-CoV-2 infection. We requested that COVID-19 should have been confirmed by a positive reverse-transcription-polymerase chain reaction test from a nasopharyngeal and/or oropharyngeal swab or from posteriori positive serological test of SARS-CoV-2 antibodies. Before starting the survey, the aim of the study and the need to electronically sign the written informed consent were explained to participants. All procedures were conducted according to the current Spanish Law on digital information (Law 14/2007 of Biomedic Investigation, Royal Decree 223/2004, General Data Protection Regulation (UE) 2016/679, and Standard Law 3/2018, December 5th, on Data Protection and Digital Rights). Participants did not receive any incentive (monetary or non-monetary) for their voluntary completion of the survey.

### 2.3. Survey

Twenty patients with post-COVID-19 condition, not included in the final sample, participated in a pilot study to evaluate the comprehensiveness of the survey. According to this pilot study, participants need 15 min to complete the survey. No question was potentially excluded.

The survey was divided into three parts. First, demographic (age, sex, nationality, marital status, educational level, occupational status, and monthly income) and clinical (pre-existing medical comorbidities diagnosed by a medical doctor) data were collected. Second, the following list of post-COVID symptoms was created: fatigue, dyspnoea, pain symptoms (generalized and headache), anosmia, ageusia, brain fog, hair loss, concentration loss, diarrhea, skin rashes, palpitations/tachycardia, ocular disorders, and cough. Further, participants were asked for the self-reported presence of depression, anxiety, or insomnia. Participants marked all symptoms that had started after COVID-19 and that persisted at the time of the survey (categorized into <5; 5–7; and >7 post-COVID symptoms).

The number of previous medical comorbidities (None; 1–2; and ≥3), alcohol consumption over the previous 30 days (Yes/No), and smoking status (smoker; ex-smoker; and non-smoker) were also collected. Finally, participants were also asked to rate their perceived health-related quality of life as “good”, “poor”, or “very poor”.

Third, medication intake was collected. We considered replies to the following two questions as dichotomous dependent variables (Yes/No):

1. *Have you taken opioid analgesics prescribed by a medical doctor within the last 30 days*? Participants answering “Yes” marked the type of opioid analgesic by using their commercial names: Tramadol, codeine, Fentanyl, Oxycodone, Morphine, Pethidine, Buprenorphine, Hydromorphone, Tapentadol, or Methadone;
2. *Have you taken non-opioid analgesics prescribed by a medical doctor within the last 30 days*? Participants answering “Yes” marked the type of non-opioid analgesic by using their commercial names, including Paracetamol, ibuprofen, metamizole, or Acetylsalicylic acid.

To also gain information about other medication use, the last question collected data about anxiolytics and antidepressant consumption in the past 30 days.

We finally asked about Cannabis, Marijuana, or Hashish consumption in the last 12 months.

### 2.4. Statistical Analysis

The Stata statistical software (Stata Corp, College Station, TX, USA. Stata/SE 16) was used for the analysis. Prevalence rates of use of opioid and non-opioid analgesics were calculated by study variables. Univariate comparison of proportions was calculated with Pearson’s χ^2^ test with statistical significance set at *p* < 0.05 (2-tailed).

To estimate the independent effect of each study variable on the consumption of opioid and non-opioid analgesics, we calculated the corresponding adjusted odds ratio (AOR) and their 95% Confidence Intervals (CI) via multivariate logistic regression analysis according to Hosmer et al. [23].

Step 1: All variables that obtained a significance less than 0.25 in the univariate analysis were selected as candidates to be in the end multivariate model.

Step 2: A multivariable model containing all covariates identified for inclusion in Step 1 was fit to assess the importance of each covariate using the *p*-value of its Wald statistic. Variables that do not contribute at traditional levels of significance were eliminated, and a new model was fit. The newer, smaller model should be compared to the old, larger model using the partial likelihood ratio test.

Step 3: For each variable removed in Step 2, we compared the values of the estimated coefficients (ORs) in the final model with those in the final model, adding this variable. Any variable that produced notable changes in the magnitude of the estimated coefficients was added back to the model, as this was important to provide a necessary adjustment for the effect of the variables that remain in the model. Steps 2 and 3 were repeated until all important variables were included in the model, and those excluded were of no clinical or statistical significance.

Step 4: Each variable not selected in Step 1 was added to the model obtained at the end of Step 3, one at a time, and their significance was checked either by the Wald statistic *p*-value or the partial likelihood ratio test if it is a categorical variable with more than 2 levels. This step is vital for identifying variables that, by themselves, are not significantly related to the outcome but make an important contribution in the presence of other variables. We refer to the model at the end of Step 4 as the preliminary main effect model.

Two models were generated, as follows: one to identify factors associated with opioid analgesic use and a second model to identify factors associated with non-opioid analgesic consumption.

## 3. Results

The descriptive characteristics of the study subjects are shown in Table 1. The sample consisted of 390 COVID-19 survivors (81.03% women, mean age: 47.60, SD: 8.75 years). The data revealed that 24.10% (*n* = 94) of subjects reported having taken opioid analgesics in the past 30 days, whereas 82.30% (*n* = 321) used non-opioid analgesics in the same period.

**Figure 1:**
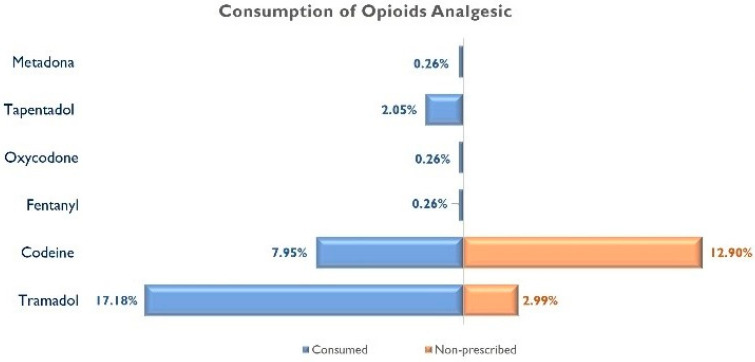
Consumption of opioid analgesics in individuals with post-COVID-19 condition.

**Figure 2:**
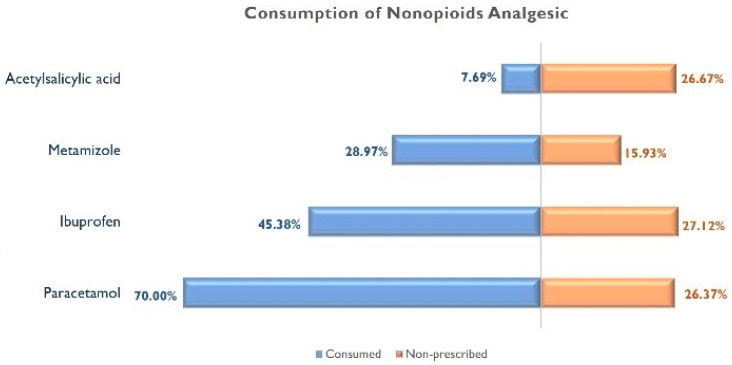
Consumption of non-opioid analgesics in individuals with post-COVID-19 condition.

Tramadol (17.18%) and codeine (7.95%) were the most commonly used opioid analgesics, whereas Paracetamol (70%) and ibuprofen (45.40%) were the most commonly used non-opioid analgesics. Figure 1 graphs the prescribed/non-prescribed consumption of opioid analgesics, whereas Figure 2 graphs the prescribed/non-prescribed consumption of non-opioid analgesics. As can be observed, almost 3% of respondents consumed Tramadol without a medical prescription, whereas the consumption of non-prescribed Paracetamol and ibuprofen reached 27% of the sample.

Table 2 summarizes prevalence data for opioid and non-opioid analgesic use according to sociodemographic variables, health profile, and use of healthcare resources. The prevalence of opioid and non-opioid analgesic consumption of respondents with post-COVID-19 condition was higher in women than men (25.95% vs. 16.22% and 84.49% vs. 72.97%, respectively). The gender difference was significant for non-opioid analgesia (*p* = 0.019). Analgesic consumption increased with age, with higher prevalence values in subjects older than 55 years old (*p* = 0.005).

Analgesic use among patients with post-COVID-19 condition who have more than seven post-COVID symptoms was 35.34% for opioid analgesics and 92.48% for non-opioid analgesics (both, *p* < 0.001). Regarding co-ingestion with other medicines, the prevalence of analgesic use (both opioid and non-opioid) was greater among the population who declared that they had consumed psychotropic medication in the previous year.

Table 3 presents multivariate logistic regression analyses on opioid/non-opioid analgesic consumption in our sample. Females were more likely to consume non-opioid analgesics (aOR 2.20, 95%CI 1.15, 4.22) than males. No significant association between gender and opioid analgesic consumption was observed (aOR 1.65, 95%CI 0.76, 3.57).

Marital status (aOR 2.96, 95%CI 1.43, 6.12), monthly income < EUR 1000 (aOR 3.81, 95%CI 1.37, 10.61), number of post-COVID symptoms (aOR 2.64, 95%CI 1.18, 5.87) and anxiolytic consumption within the past 30 days (aOR 1.85, 95%CI 1.05, 3.25) were independently and significantly associated with a greater likelihood of opioid analgesic consumption (Table 3). An age older than 55 years old (aOR 3.30, 95%CI 1.34, 8.09) and anxiolytic consumption within the past 30 days (aOR 2.61, 95%CI 1.36, 4.98) act as significant predictors for non-opioid analgesic use (Table 3). Opioid analgesic consumption in the past 30 days was the variable with the strongest association (aOR 3.41, 95%CI 1.27, 6.11) with non-opioid analgesic consumption (Table 3).

## 4. Discussion

Although several studies have investigated analgesic consumption during the COVID-19 pandemic [16,19,24,25,26,27,28], they provide inconclusive evidence of the effect of COVID-19 on pain management. We analyzed opioid and non-opioid analgesic consumption patterns in a cohort of individuals with post-COVID-19 condition.

Opioids are a relatively inexpensive and effective approach for individuals with chronic pain. Although opioid analgesic consumption was high in the 1990s, its use has decreased in most European countries over the past decades [29]. Nevertheless, opioid consumption is country-dependent. For instance, prescription opioid use has increased in Spain over the last decade [20], but compared with data from the USA or Canada [30,31], it is still significantly lower. Thus, opioid analgesic consumption within the general population has also increased in the last decade in other European countries, such as the Netherlands and the Nordic countries [32,33]. Our study showed a prevalence of opioid analgesic use of 24.10% in patients with post-COVID-19 condition. The COVID-19 pandemic altered the practice and prescribing patterns of pain physicians. Joyce et al. showed that physicians were those healthcare professionals who changed their prescribing patterns by increasing their prescription of opioids by up to 28.8% and their prescription of non-opioid analgesics by up to 26.2% [18].

Tramadol and codeine were the opioids most frequently consumed by patients with post-COVID-19 condition in the current study, in agreement with consumption data for these substances in the USA and other European countries [33,34,35,36]. Tramadol prescription rates have continuously increased both nationally and throughout all US regions [36]. In fact, Tramadol is the most used active ingredient among all opioid analgesics in Spain [20]. Thus, Tramadol was the opioid analgesic most commonly used without a medical prescription. It should be considered that the most common source for opioid misuse consists of prescriptions or recommendations from relatives and friends. Opioids prevail in homes in our communities, and it may be relatives who are insufficiently conscious of the risks of misuse who are enabling their use by other family members at home.

There is an increasing concern about the misuse of prescribed and over-the-counter (OTC) codeine. A survey on the use of OTC medicines containing codeine reported that 6% of Irish, 13% of South African, and 16% of English people reported having purchased OTC medicines containing codeine on a weekly basis [37].

Frequent non-opioid analgesic use seems to be justified and necessary in most cases due to intense and frequent pain symptoms. The prevalence of non-opioid use in our patients was 82.30%. Common non-opioid analgesic and nonsteroidal anti-inflammatory drugs are the most commonly used drugs for headaches in the acute phase of COVID-19 [5,38]. Thus, Paracetamol (70%) and ibuprofen (45.38%) were the non-opioid analgesics most frequently used by individuals with post-COVID-19 condition in our study. Interestingly, internet search interest for ibuprofen and acetaminophen increased during the COVID-19 pandemic in the USA [17]. Nino-Orrego et al., in a study aiming to know the drug therapies offered by community pharmacies for the prevention and treatment of COVID-19, found that acetaminophen, ibuprofen, and acetylsalicylic acid were the most recommended medications [39]. A recent cross-sectional study conducted in Spain reported that 95% of patients used acetaminophen (93%), ibuprofen (17%), and metamizole (12%) as analgesics for their headaches [38].

Overall, it is generally assumed that women exhibit higher consumption rates of non-opioid analgesics than men [40,41]. The prevalence of analgesic consumption was higher among the female population in our study, although sex differences were only significant for non-opioid analgesia (*p* = 0.019). Sex differences in analgesic prescription are not merely the result of higher prevalence rates of pain in women when compared with men since this therapeutic variability is also related to factors such as educational level or social class. Chilet-Rosell et al., using data from the Spanish National Health Survey, showed that women were more likely to be prescribed analgesics than men (OR 1.74) [42]. Similarly, our results show that women are twice as likely to use non-opioid analgesics than men (aOR 2.20, 95%CI 1.15, 4.22).

We observed that subjects aged > 40 years were almost three times more likely to use non-opioid analgesics than younger individuals, in agreement with previous studies. The Tromsø Study showed a lower non-opioid analgesic consumption within the Norwegian population aged <45 years compared to those older than 45 years of age [43]. A cross-sectional population-based study using data from the Brazilian National Survey on Access, Use, and Promotion of Rational Use of Medicines also found that the prevalence of non-opioid analgesic use was higher among individuals aged 60 years and over [44]. When variable monthly income was analyzed, the results showed that individuals earning less than EUR 1000 per month were more likely to take opioid analgesics (aOR 3.81, 95%CI 1.37, 10.61). These data are consistent with those from a recent USA study, which found that individuals with a lower income are more likely to be exposed to prescription opioid analgesics than those with a higher monetary income [45].

Patients with post-COVID-19 condition in our study who reported a greater number of post-COVID symptoms exhibited a greater probability of consuming opioid analgesics (aOR 2.64, 95% CI 1.18, 5.87). Pain is a common post-COVID symptom. Fernández-de-las-Peñas et al. found that musculoskeletal post-COVID pain is present in up to 47.7% of previously hospitalized COVID-19 survivors [46]. Al-Aly et al., in an investigation into the characterization of post-acute sequelae of COVID-19, identified incident sequelae such as musculoskeletal pain and showed an increased incident use of several therapeutic approaches, including pain medications (opioids and non-opioids) [47]. Scherrer et al., in a prospective cohort study of non-cancer pain during the COVID-19 pandemic in the USA, showed that prescription opioid use in the prior three months was associated with moderate stress due to COVID-19 [48].

Sedative-hypnotic use is also common among individuals using opioid analgesics in our study. When we analyzed the use of psychoactive substances among our study population, we found that anxiolytic consumption during the last 12 months behaved as a predictor of opioid analgesic use (aOR 1.85, 95%CI 1.05, 3.25) and non-opioid analgesic consumption (aOR 2.61, 95%CI 1.36, 4.98). Tubbs et al., using data from the U.S. National Survey on Drug Use and Health for 2015–2018, also showed that people who had used opioids in the last year were four times more likely to consume benzodiazepines (OR4.40, 95%CI 3.61, 5.4) [49]. Prescription of anxiolytics has increased due to an increased prevalence of psychological disturbances, e.g., anxiety and depression, during the COVID-19 pandemic [50]. Czeisler et al. observed a 33% increase in the prevalence of anxiety and depression among the US adult population, infected or not [51]. Thus, the prevalence of anxiety and depressive disorders among COVID-19 individuals is also higher than in those without COVID-19 [52].

Finally, our outcomes indicate that among patients with post-COVID-19 condition, subjects who consumed non-opioid analgesics were three times more likely to have consumed opioid analgesics in the past month (aOR 3.41, 95% CI 1.27, 6.11). The coronavirus pandemic changed the prescribing patterns of pain physicians who had increased their prescription of opioid and non-opioid analgesics [18]. Thus, there is evidence of possible inappropriate use of these medicines, including the use of multiple analgesics, as shown by the study of da Silva Dal Pizzol et al., where the use of multiple analgesics (two or three) was observed in up to 20% of the analgesic users [44]. Opioid abuse is a risk factor for mortality amongst hospitalized COVID-19 patients. Hence, it is critical to educate society about the consequences of unauthorized opium consumption. Ostinelli et al. synthesized the best available recommendations on the management and care of individuals at risk of substance use disorders during the COVID-19 pandemic and observed that current guidance does not provide advice on service access strategies tailored to individuals with substance use disorders [53].

Although this study represents the first step in identifying factors associated with opioid and non-opioid analgesic consumption in patients suffering from post-COVID-19 condition, some limitations should be recognized. First, it is known that respondents with more severe post-COVID-19 condition have a higher tendency to actively participate in surveys. In fact, patients with more severe symptomatology (which may be associated with a higher analgesic consumption) are those participating more actively in survey studies. Thus, the observed prevalence rates of opioid analgesic consumption could be overestimated, although our data were similar to those reported in previous studies. Therefore, population-based surveys are needed to extrapolate current results. In fact, prevalence rates observed in our study should not be extrapolated to the general population since our data are valid in our sample and its circumstances. Second, the cross-sectional study design (an inherent limitation of surveys on medication consumption) does not permit the determination of cause-and-effect associations. Longitudinal studies collecting data at different follow-up periods would permit the identification of prolonged consumption of these medications.

Third, all data were self-reported; accordingly, the current data about opioid and non-opioid analgesic consumption may be underestimated because of potential sociocultural beliefs surrounding psychotropic medication use.

## 5. Conclusions

The prevalence of opioid and non-opioid analgesic consumption in respondents to this survey with post-COVID-19 condition was 24.1% and 82.3%, respectively. Women with post-COVID-19 condition self-reported higher non-opioid analgesic consumption than men with post-COVID-19 condition. Married/coupled marital status, lower monthly income, number of post-COVID symptoms, and anxiolytic consumption were risk factors associated with opioid consumption. Older age and anxiolytic consumption were factors associated with a higher probability of non-opioid consumption. Our data have important implications for public health since they offer scientific evidence concerning opioid and non-opioid analgesic consumption among subjects with post-COVID-19 condition. Understanding opioid and non-opioid analgesics is critical, as this will facilitate decision-making for greater control in their prescription by healthcare professionals, particularly the use of opioid analgesics, to avoid inappropriate use of these drugs and prevent subsequent harm to non-opioid analgesic users [54].

## Figures and Tables

**Table 1:** Sample characteristics of total survey sample.

	N	%
**Gender**		
Male	74	18.97
Female	316	81.03
**Age group**		
<40 years	66	17.28
40–55 years	242	63.35
>55 years	74	19.37
**Nationality**		
Immigrants	6	1.54
Spanish	384	98.46
**Marital status**		
Single	121	31.03
Married or couple	208	53.33
Divorced/widow	54	13.85
**Educational level**		
Primary school	18	4.62
Secondary school	111	28.46
Higher education	261	66.92
**Occupational status**		
Employed	202	51.79
Unemployed	41	10.51
Inactive	95	24.36
**Monthly income**		
<EUR 1000	29	7.44
EUR 1000–EUR 2000	129	33.08
>EUR 2000	189	48.46
**Alcohol consumption in the past 12 months**		
No	189	48.46
Yes	201	51.54
**Smoking habit in the past 12 months**		
Smoker	35	8.97
Ex-smoker	164	42.05
Non-smoker	191	48.97
**Pain symptoms (generalized, headache, and chest pain)**		
No	28	7.18
Yes	362	92.82
**Number of chronic conditions**		
0	111	28.46
1–2	121	31.03
≥3	158	40.51
**Number of post-COVID symptoms**		
<5	86	22.05
5–7	171	43.85
>7	133	34.10
**Self-assessment of health status**		
Good	106	27.18
Poor	173	44.36
Very poor	111	28.46
**Hospitalization in preceding 12 months**		
No	283	72.56
Yes	107	27.44
**Admission to intensive care in preceding 12 months**		
No	376	96.41
Yes	14	3.59
**Opioid Analgesic consumption in the past 30 days**		
No	296	75.90
Yes	94	24.10
**Non-opioid analgesics consumption in the past 30 days**		
No	69	17.69
Yes	321	82.31
**Anxiolytic consumption in the past 30 days**		
No	215	55.13
Yes	175	44.87
**Antidepressant consumption in the past 30 days**		
No	237	60.77
Yes	153	39.23
**Cannabis, Marijuana, or Hashish consumption in the last 12 months**		
No	374	95.9
Yes	16	4.10

**Table 2:** Prevalence of opioid and non-opioid analgesic consumption in patients with post-COVID-19 condition by sociodemographic, health profile, and use of healthcare resource variables.

	Opioid Analgesic			Non-Opioid Analgesic		
	N	% (95% CI)	*p* Value	N	% (95% CI)	* *p* Value
**Gender**						
Male	12	16.22 (9.18, 25.83)	0.078	54	72.97 (62.12, 82.07)	0.019 *
Female	82	25.95 (21.35, 30.99)		267	84.49 (80.20, 88.16)	
**Age group**						
< 40 years	9	13.64 (6.97, 23.41)	0.095	45	68.18 (56.35, 78.46)	0.005 *
40–55 years	64	26.45 (21.19, 32.26)		206	85.12 (80.23, 89.18)	
>55 years	18	24.32 (15.66, 34.95)		63	85.14 (75.75, 91.84)	
**Nationality**						
Immigrants	1	16.67 (1.86, 55.81)	0.668	6	100 (100.00, 100.00)	0.252
Spanish	93	24.22 (20.14, 28.69)		315	82.03 (77.96, 85.62)	
**Marital status**						
Single	16	13.22 (8.08, 20.10)	0.005 *	90	74.38 (66.09, 81.52)	0.036 *
Married or couple	59	28.37 (22.57, 34.76)		178	85.58 (80.32, 89.85)	
Divorced/widow	18	33.33 (21.88, 46.52)		46	85.19 (73.98, 92.74)	
**Educational level**						
Primary school	4	22.22 (8.00, 44.58)	0.000 *	14	77.78 (55.42, 92.00)	0.144
Secondary school	42	37.84 (29.22, 47.08)		98	88.29 (81.34, 93.27)	
Higher education	48	18.39 (14.05, 23.43)		209	80.08 (74.92, 84.58)	
**Occupational status**						
Employed	44	21.78 (16.52, 27.85)	0.243	165	81.68 (75.92, 86.55)	0.786
Unemployed	10	24.39 (13.29, 38.97)		34	82.93 (69.38, 92.02)	
Inactive	30	31.58 (22.89, 41.37)		81	85.26 (77.12, 91.30)	
**Monthly income**						
<EUR 1000	12	41.38 (24.97, 59.41)	0.001 *	26	89.66 (74.90, 97.00)	0.652
EUR 1000–EUR 2000	43	33.33 (25.64, 41.77)		107	82.95 (75.76, 88.67)	
>EUR 2000	32	16.93 (12.11, 22.76)		152	80.42 (74.33, 85.59)	
**Alcohol consumption in the past 12 months**						
No	40	21.16 (15.81, 27.40)	0.188	151	79.89 (73.75, 85.13)	0.226
Yes	54	26.87 (21.1, 33.29)		170	84.58 (79.11, 89.06)	
**Smoking habit in the past 12 months**						
Smoker	7	20.00 (9.42, 35.31)	0.199	29	82.86 (68.03, 92.51)	0.077
Ex-smoker	47	28.66 (22.16, 35.91)		143	87.20 (81.44, 91.65)	
Non-smoker	40	20.94 (15.63, 27.12)		149	78.01 (71.74, 83.44)	
**Pain symptoms (generalized, headache, and chest pain)**						
No	5	17.86 (7.16, 34.80)	0.422	18	64.29 (45.84, 79.94)	0.009 *
Yes	89	24.59 (20.36, 29.21)		303	83.7 (79.64, 87.23)	
**Number of chronic conditions**						
None	22	19.82 (13.23, 27.96)	0.377	87	78.38 (70.05, 85.25)	0.002 *
1–2	29	23.97 (17.04, 32.13)		91	75.21 (66.98, 82.24)	
≥3	43	27.22 (20.73, 34.52)		143	90.51 (85.20, 94.35)	
**Number of post-COVID symptoms**						
<5	12	13.95 (7.86, 22.42)	0.000 *	69	80.23 (70.90, 87.57)	0.000 *
5–7	35	20.47 (14.95, 26.98)		129	75.44 (68.60, 81.43)	
>7	47	35.34 (27.60, 43.71)		123	92.48 (87.07, 96.07)	
**Self-assessment of health status**						
Good	20	18.87 (12.31, 27.10)	0.229	86	81.13 (72.90, 87.69)	0.018
Poor	42	24.28 (18.35, 31.06)		152	87.86 (82.38, 92.09)	
Very poor	32	28.83 (21.03, 37.72)		83	74.77 (66.13, 82.15)	
**Hospitalization in preceding 12 months**						
No	65	22.97 (18.36, 28.13)	0.394	229	80.92 (76.04, 85.17)	0.242
Yes	29	27.10 (19.37, 36.05)		92	85.98 (78.48, 91.57)	
**Admission to intensive care in preceding 12 months**						
No	91	24.20 (20.08, 28.72)	0.812	309	82.18 (78.07, 85.79)	0.734
Yes	3	21.43 (6.43, 46.90)		12	85.71 (61.51, 96.91)	
**Opioid Analgesic consumption in the past 30 days**						
No	N.A.			232	78.38 (73.43, 82.78)	0.000 *
Yes				89	94.68 (88.74, 97.94)	
**Non-opioid analgesic consumption in the past 30 days**						
No	5	7.25 (2.82, 15.15)	0.000 *	N.A.		
Yes	89	27.73 (23.04, 32.81)				
**Anxiolytic consumption in the past 30 days**						
No	35	16.28 (11.81, 21.65)	0.000 *	162	75.35 (69.28, 80.74)	0.000 *
Yes	59	33.71 (27.02, 40.94)		159	90.86 (85.92, 94.46)	
**Antidepressant consumption in the past 30 days**						
No	48	20.25 (15.52, 25.71)	0.027 *	185	78.06 (72.47, 82.97)	0.006 *
Yes	46	30.07 (23.22, 37.65)		136	88.89 (83.19, 93.14)	
**Cannabis, Marijuana, or Hashish consumption in the last 12 months**						
No	90	24.06 (19.94, 28.59)	0.932	308	82.35 (78.25, 85.96)	0.916
Yes	4	25.00 (9.08, 49.07)		13	81.25 (57.92, 94.42)	

* *p*-value: Statistically significant differences (*p* < 0.05). N.A.: not applicable; CI: confidence interval

**Table 3:** Multivariable logistic regression analysis of factors associated with opioid and non-opioid analgesics consumption in patients with post-COVID-19 condition.

	Opioid Analgesic	Non-Opioid Analgesic
	**aOR (95% CI)**	**aOR (95% CI)**
**Gender**		
Male	Reference	Reference
Female	1.65 (0.76, 3.57)	2.20 (1.15, 4.22)
**Age group**		
<40 years	Reference	Reference
40–55 years	1.84 (0.77, 4.39)	3.30 (1.65, 6.60)
>55 years	1.49 (0.54, 4.12)	3.30 (1.34, 8.09)
**Marital status**		
Single	Reference	Reference
Married or couple	2.96 (1.43, 6.12)	N.S.
Divorced/widow	2.13 (0.86, 5.28)	N.S.
**Monthly income**		
>EUR 2000	Reference	Reference
EUR 1000–EUR 2000	2.90 (1.59, 5.26)	N.S.
<EUR 1000	3.81 (1.37, 10.61)	N.S.
**Number of post-COVID symptoms**		
<5	Reference	Reference
5–7	1.36 (0.62, 2.97)	N.S.
>7	2.64 (1.18, 5.87)	N.S.
**Anxiolytic consumption in the past 30 days**		
No	Reference	Reference
Yes	1.85 (1.05, 3.25)	2.61 (1.36, 4.98)
**Opioid Analgesic consumption in the past 30 days**		
No	N.A.	Reference
Yes		3.41 (1.27, 6.11)
**Non-opioid analgesic consumption in the past 30 days**		
No	Reference	N.A.
Yes	2.70 (0.99, 7.35)	

aOR: adjusted odds ratio; CI: Confidence Interval; N.S.: non-significant association; and N.A.: not applicable.

## Data Availability

All data relevant to the study are included in the article.
